# Colorectal Cancer Awareness Among the General Population in Northern Saudi Arabia

**DOI:** 10.7759/cureus.58724

**Published:** 2024-04-22

**Authors:** Mohamed M Abd El Mawgod, Abdullah S Alanazi, Nawaf S Alenezi, Mugrin R Alrwaili, Saleh I Alsuqub

**Affiliations:** 1 Family and Community Medicine, College of Medicine, Northern Border University, Arar, SAU; 2 Public Health and Community Medicine, Faculty of Medicine, Al-Azhar University, Asyut, EGY; 3 College of Medicine, Northern Border University, Arar, SAU

**Keywords:** risk factors, signs, symptoms, awareness, colorectal cancer

## Abstract

Background: One of the most prevalent types of cancer is colorectal cancer (CRC). Increased consumption of foods derived from animals, smoking, and family history are all regarded as CRC risk factors. A significant obstacle to the use of screening programs for CRC is community unawareness.

Objective: This study aimed to assess the awareness of symptoms, signs, and screening modalities of CRC among the adult population in Arar City, Northern Saudi Arabia.

Subjects and methods: A cross-sectional study was conducted among males and females living in Arar City who were 18 years of age or older.

Results: More than half (56.6%; 233) of the participants correctly identified that the colon is the large intestine, 61.7% (254) identified that the rectum is the distal part of it, and nearly a fifth stated that the function of the colon is water reabsorption. The majority stated abdominal pain (78.2%; 322) and change in bowel habits (76%; 313) are symptoms of CRC, but less than 60% (240) reported nausea and vomiting. Concerning participants' risk factor awareness of CRC, smoking is most frequently cited, followed by irritable bowel syndrome (IBS), fatty food, family history, and colon polyps.

Conclusion: Although not perfect, the current analysis demonstrates that there is accepted public awareness about CRC. We recommend the adoption of education initiatives via mass media and at regular religious events like Jumma to increase community awareness and knowledge.

## Introduction

Worldwide, including in Saudi Arabia, colorectal cancer (CRC) is one of the most prevalent cancers [1,2]. For Saudi men and women, CRC came in first and third, respectively. In 2018, Saudi nationals experienced 1,908 new diagnoses of CRC, representing 12.2% of all newly diagnosed cancer cases [3]. The estimated number of new cases of CRC globally was 1.9 million in 2020, and there were 935,000 fatalities related to it [4]. The Colorectal Cancer Early Detection Initiative, which focuses on people 50 years of age or older, was introduced by the Saudi Ministry of Health in 2017 [5].

A study carried out in Mekkah and Jeddah, Saudi Arabia, showed that more than half of the participants (58% and 53%) had inadequate knowledge of the methods for identifying CRC and the risk factors associated with it [6]. Additionally, in Jeddah, Imran et al. revealed that 82.3% of the participants knew about CRC and that 56.8% believed it could be managed effectively. Furthermore, of the participants, as many as 60% knew about the symptoms, signs, and risk factors related to the disease [7]. Al-Hajeili et al. also discovered in Jeddah that only educational level and family history of CRC were significant predictors of awareness of CRC screening and gender was found to be a major predictor of knowledge levels [8]. According to the Akanbi et al. study, North and Central Asian countries have inadequate knowledge of CRC symptoms and insufficient awareness of the availability of CRC screening [9].

In Western Saudi Arabia, Al-Thafar et al. found that 39% of males and 42% of females were unaware of CRC screening tests [10]. Al-Sharif et al., in their survey, stated that in the Asir region of Saudi Arabia, approximately one-third of respondents correctly identified the function of both the colon and rectum; 71.6% knew what a colon or rectum is; around 22% were aware of the precise incidence of CRC; and 23% correctly recognized the timing of CRC testing [11]. In their study of more than 4000 people from everywhere in Saudi Arabia, Alkhayyat et al. discovered that 44% of respondents believed that an important indicator linked to CRC was unexplained weight loss and approximately 61% were unfamiliar with CRC screening methods [12].

Alotaibi et al. conducted another national survey in Saudi Arabia and found that of the 521 participants, around half had a low level of awareness of the symptoms and risk factors associated with CRC [13]. Furthermore, a similar national survey indicated that more than 50% of the respondents were knowledgeable of CRC [14]. According to the study by Alshaer et al., Saudi Arabian participants had insufficient knowledge and poor awareness of CRC [15]. To our knowledge, there has been no study conducted in Saudi Arabia's northern region regarding CRC awareness. The current research aims to assess the adult public's awareness of the symptoms, signs, and screening modalities of CRC in Northern Saudi Arabia.

## Materials and methods

Study design and setting

A cross-sectional study was conducted from April 1 to October 31, 2023. Males and females living in Arar City who were 18 years of age or older made up the study sample population.

Inclusion and exclusion criteria

The study only included participants who were willing to take part and who were older than 18. In contrast, participants who declined or were less than 18 were not included.

Ethical clearance

The Local Committee of Bioethics (HAP-09-043) of Northern Border University approved the study prior to the data collection (approval number: 30/44/H dated April 26, 2023). Individuals gave their informed consent, and participant confidentiality was maintained. 

Methodology

The study used a structured and validated questionnaire prepared by reviewing the relevant literature. The questionnaire was reviewed and approved by two physicians (family and gastroenterology). A pilot study on 20 subjects before the study was conducted to eliminate any ambiguities related to the questionnaire (their findings were not involved in the analysis).

There were five domains in the questionnaire: (1) Socio-demographic data was included in the first domain (age, gender, marital status, educational level, occupation smoking habits, and body mass index). (2) Background data on the colon and rectum made up the second domain (definition and function of both the colon and rectum and prevalence of CRC). (3) The third domain had questions about CRC signs and symptoms (abdominal pain, change in bowel habits, blood in stool, nausea and vomiting, and yellow coloration of the skin and eye). (4) CRC risk factor-related questions were included in the fourth domain such as smoking, irritable bowel syndrome (IBS), fatty meals, family history, and colon polyps. (5) The fifth domain included questions about the diagnostic modalities for CRC (colonoscopy, CT, fecal occult blood, ultrasound, X-ray, and the possibility of cure from CRC).

The sample size was calculated according to the following formula: N = (Z1-α/2) 2 P (1-P)/d2 = (1.96)2 × 0.52 × (0.48)/(0.5)2 = 383.4 ~ 384. Here, (Z1−α/2) is the standard normal variate at 5% type 1 error (1.96); (P) is the expected proportion of awareness in Saudi Arabia (52%) [12]; and (d) is the absolute error (0.05). The sample was completed at 412.

Following ethical approval and after clarifying the objectives of the study and obtaining written informed consent (an agreement for participation was placed at the beginning of the questionnaire), we used an online technique for gathering data from participants using a variety of social media platforms (WhatsApp, Facebook, and Telegram).

Data analysis

After being verified for accuracy and completeness, the obtained data was coded and entered using IBM SPSS Statistics for Windows, Version 23.0 (Released 2015; IBM Corp., Armonk, New York, United States). Frequency and percentage were reported for the qualitative data, and the chi-squared test was used as statistical significance. The mean and standard deviation were used to present quantitative data. The statistical significance level was set at 5%.

## Results

A total of 412 subjects took part in the study. Around 50% (207) were males, and their mean age was 33±12. More than half were in the age group of 20-40 years, slightly less than half (48.8%; 201) were married, the majority (76.5%; 315) had graduated from university, and 54% (223) were unemployed. Less than a fifth of participants are smokers, while 25% (103) of them are obese, as shown in Table 1.

**Table 1:** Socio-demographic characteristics of the studied participants

Items	No (412)	%
Age		
Less than 20 years	54	13.1
20-40 years	231	56.1
More than 40 years	127	30.8
Gender		
Male	207	50.2
Female	205	49.8
Marital status		
Married	201	48.8
Single	193	46.8
Divorced	13	3.2
Widowed	5	1.2
Educational level		
Intermediate	10	2.4
Secondary	63	15.3
University	315	76.5
Postgraduate	24	5.8
Occupation		
Not employed	223	54.1
Government employed	130	31.7
Private job	17	4.1
Free jobs	9	2.2
Retired	33	8
Smoking habits		
Yes	74	18
No	311	75.5
Ex-smoker	27	6.5
Body mass index		
Underweight	21	5.1
Ideal	153	37.1
Overweight	135	32.8
Obese	103	25

Table 2 provides the participants' information about the knowledge related to the colon and rectum. More than half (56.6%; 233) of the participants correctly identified that the colon is the large intestine, 61.7% (254) identified that the rectum is the distal part of it, nearly a fifth of them stated that the function of the colon is water reabsorption, and slightly less than a fifth declared that the prevalence of the disease is high. This shows that, while not perfect, the general level of public awareness is acceptable.

**Table 2:** Background information on the colon and rectum of the study subjects CRC: colorectal cancer

Items	No	%
In your opinion, what is the colon?		
The large intestine	233	56.6
The small intestine	29	7
The stomach	30	7.3
The stomach and small intestine	66	16
I don't know	54	13.1
In your opinion, what is the rectum?		
The last part of the stomach	38	9.2
The last part of the small intestine	58	14.1
The last part of the large intestine	254	61.7
I don't know	62	15
In your opinion, what is the function of the colon?		
Digestion of food	86	20.9
Waste storage	132	32
Water reabsorption	88	21.4
Does not have a function	17	4.1
I don't know	89	21.6
In your opinion, what is the prevalence of CRC?		
High	76	18.4
Moderate	255	61.9
Rare	81	19.7

The participants' awareness of CRC symptoms and signs is shown in Table 3. The majority, 78.2% (322), stated abdominal pain and 76% (313) bowel habit changes, and less than 60% (240) reported nausea and vomiting for CRC symptoms. Less than 40% of the respondents indicated that yellow discoloration of the eye and skin is a sign of CRC, whereas the majority thought the presence of blood in the stool.

**Table 3:** Participants' awareness of signs and symptoms of CRC CRC: colorectal cancer

Items	No	%
Abdominal pain is one of the symptoms of CRC		
Yes	322	78.2
No	20	4.9
I don't know	70	17
Bowel habit change is one of the symptoms of CRC		
Yes	313	76
No	13	3.2
I don't know	86	20.8
Blood in stool is one of the signs of CRC		
Yes	295	71.6
No	34	8.3
I don't know	83	20.1
Nausea and vomiting are some of the symptoms of CRC		
Yes	240	58.3
No	64	15.5
I don't know	108	26.2
Yellow discoloration of the eye and skin is one of the signs of CRC		
Yes	153	37.2
No	104	25.2
I don't know	155	37.6

Table 4 displays the respondents' knowledge of CRC risk factors. More than two-thirds reported smoking and IBS; more than half mentioned a fatty diet; and less than 50% cited family history and colon polyps.

**Table 4:** Participants' knowledge of CRC risk factors CRC: colorectal cancer; IBS: irritable bowel syndrome

Items	No	%
In your opinion, is smoking a risk factor of CRC?		
Yes	284	68.9
No	50	12.1
I don't know	78	18.9
In your opinion, is IBS a risk factor of CRC?		
Yes	278	67.5
No	33	8
I don't know	101	24.5
In your opinion, is fatty food a risk factor of CRC?		
Yes	229	55.6
No	65	15.8
I don't know	118	28.6
In your opinion, is a family history of CRC a risk for its development?		
Yes	179	43.4
No	174	42.2
I don't know	59	14.3
In your opinion, are colon polyps a risk factor of CRC?		
Yes	165	40
No	57	13.8
I don't know	190	46.1

Table 5 demonstrates the participants' awareness of CRC diagnostic methods. Most respondents mentioned that colonoscopy can identify CRC; slightly more than 60% (252) reported a CT scan; slightly less than 60% (239) reported fecal occult blood; and roughly 40% (168) mentioned an ultrasound and an X-ray. More than two-thirds believed that CRC is curable; slightly less than 30, 28.7% (118), mentioned that the best time to start screening is at the age of 50 years.

**Table 5:** Distribution of participants' awareness of CRC diagnostic methods CRC: colorectal cancer; IBS: irritable bowel syndrome

Items	No	%
In your opinion, can colonoscopy be used for the diagnosis of CRC?		
Yes	361	87.6
No	11	2.7
I don't know	40	9.7
In your opinion, can a CT scan be used for the diagnosis of CRC?		
Yes	252	61.2
No	63	15.3
I don't know	97	23.5
In your opinion, can fecal occult blood be used for the diagnosis of CRC?		
Yes	239	58
No	62	15
I don't know	111	27
In your opinion, can ultrasound be used for the diagnosis of CRC?		
Yes	168	40.8
No	120	29.1
I don't know	124	30.1
In your opinion, can an X-ray be used for the diagnosis of CRC?		
Yes	162	39.3
No	139	33.7
I don't know	111	27
Can CRC accruable disease be used for the diagnosis of CRC?		
Yes	286	69.4
No	13	3.2
I don't know	113	27.4
What is the suitable time for CRC screening?		
At the onset of symptoms	218	52.9
At the age of 20 years	76	18.4
At the age of 50 years and more	118	28.7
Is there a relationship between CRC and IBS?		
Yes	160	38.8
No	76	18.4
I don't know	176	42.7

Table 6 shows the gender differences in the individuals' knowledge of the risk factors for CRC. When compared to females, males are insignificantly more aware that smoking and family history are risk factors for the illness. Although the differences are statistically insignificant, females are more aware than males that colon polyps, BS, and family history are risk factors for the disease. Males are significantly (P=0.01) more aware, when compared to females, that abdominal pain is one of the symptoms of CRC. Females are significantly more aware than males of the symptoms of CRC, including nausea and vomiting (P=0.02) and yellow discoloration (P=0.003). Females are insignificantly more aware than males of the symptoms of CRC, including changes in bowel habits (P=0.08) and blood in stool (P=0.2). Insignificantly, females are more aware than males of CRC signs, such as blood in the stool (P=0.2) and altered bowel habits (P=0.08).

**Table 6:** Frequency distribution of CRC risk factor awareness by gender χ2 * test CRC: colorectal cancer; IBS: irritable bowel syndrome

Variables		Males (no=207) No (%)	Females (no=205) No (%)	P-value *
Smoking	Yes	150 (72.5)	134 (65.4)	0.2
	No	22 (10.6)	28 (13.7)	
	I don't know	35 (16.9)	43 (21)	
IBS	Yes	136 (65.7)	142 (69.3)	0.6
	No	19 (9.2)	14 (6.8)	
	I don't know	52 (25.1)	49 (23.9)	
Family history	Yes	97 (46.9)	82 (40)	0.3
	No	82 (39.6)	92 (44.9)	
	I don't know	28 (13.5)	31 (15.1)	
Fatty diet	Yes	117 (56.5)	112 (54.6)	0.09
	No	25 (12.1)	40 (19.5)	
	I don't know	65 (31.4)	53 (25.9)	
Colon polyps	Yes	80 (38.7)	85 (41.5)	0.4
	No	33 (15.9)	24 (11.7)	
	I don't know	94 (45.4)	96 (46.8)	
Symptoms				
Abdominal pain	Yes	172 (83.1)	150 (73.2)	0.01
	No	11 (5.3)	9 (4.4)	
	I don't know	24 (11.6)	46 (22.4)	
Change in bowel habit	Yes	150 (75.5)	163 (79.5)	0.08
	No	10 (4.8)	3 (1.5)	
	I don't know	48 (22.7)	39 (19)	
Nausea and vomiting	Yes	115 (55.6)	125 (61)	0.02
	No	42 (20.2)	22 (10.7)	
	I don't know	50 (24.2)	58 (28.3)	
Yellow discoloration	Yes	64 (30.9)	89 (43.5)	0.003
	No	66 (31.9)	38 (18.5)	
	I don't know	77 (37.2)	78 (38)	
Blood in stool	Yes	141 (68.1)	154 (75.2)	0.2
	No	20 (9.7)	14 (6.8)	
	I don't know	46 (22.2)	37 (18)	

## Discussion

To our knowledge, no other study has assessed a comparable topic in this region. According to the study, more than half of the respondents correctly identified the colon and the rectum; over one-fifth of them knew the function of the colon; and just under one-fifth indicated that the prevalence of CRC is high. This shows that, while not perfect, the general level of public awareness is acceptable.

Similar findings were reported by Alaqel et al. [16] in Riyadh, Saudi Arabia, who found that 51.7%, 57%, and 21.3% of the participants were aware that the colon is the large intestine, the rectum is its distal portion, and the colon's primary function is the absorption of water. Also, Galal et al. indicated that around 66.4% of their participants had poor awareness [17]. Additionally, research conducted in an Asir region in Southern Saudi Arabia revealed that 51.1%, 71.6%, 33.8%, 22.5%, and 22.1% of the participants correctly identified the site of the colon, the site of the rectum, the function of the colon, and that the incidence of CRC is high [11]. Additionally, Imran et al., in similar research, mentioned that 46.7% of the participants answered that the prevalence of CRC is high [7].

Regarding the participants' awareness of CRC symptoms, the most commonly cited symptom is abdominal pain, followed by changes in bowel habits, blood in the stool, nausea and vomiting, and yellowish skin discolorations. In a comparable study conducted in Saudi Arabia, the presence of blood in the stool was found most frequently (51.9%), followed by abdominal discomfort (51.5%), changes in bowel habits (45.7%), and nausea and vomiting (24.5%) [16]. A similar study conducted in Saudi Arabia revealed that the most commonly reported symptom was the presence of blood in the stool, followed by changes in bowel habits and abdominal pain [18]. In terms of participants' knowledge of CRC risk factors, most of them state smoking, followed by IBS, fatty food, family history, and colon polyps. 

In agreement with similar studies conducted in Riyadh by Galal et al. [17] and the western region of Saudi Arabia by Alsmkari et al. [19], smoking was the most frequent response (61.5% and 55.1%, respectively). Furthermore, family history and smoking (50.3% and 40.4%) were the most frequent responses in the study conducted by Alaqel et al. in Riyadh [16]. IBS was viewed as a risk factor by a substantial percentage of those surveyed (40.6%) in the study by Ahmed and Alrashidi [20].

In line with Alaqel et al. [16] and Galal et al. studies in Saudi Arabia, family history was reported by more than 40% of participants [17]. IBS was viewed as a risk factor by a significant percentage of respondents (40.6%) in the study by Ahmed and Alrashidi [20]. Poor diet was cited as a risk factor for CRC most frequently (54.2%), followed by IBD (50.8%), family history (37.6%), and smoking (35.3%), according to a study done in Riyadh, Saudi Arabia [21]. Once more, a reported family history of CRC (77.59%) was regarded as a risk factor for CRC in the western area of Saudi Arabia. Additionally, food and smoking were mentioned as risk factors by approximately 61%, while 45% mentioned IBD [6]. In the western region of Saudi Arabia, a little over two-thirds of respondents reported a low-fiber diet and smoking as risk factors for CRC in a study by Alnuwaysir et al. [22]. Alsayed et al. in Al-Madinah Al-Monawarrah, Saudi Arabia, indicated that 35.5%, 62.2%, and 57.1% of respondents reported that family history, smoking, and colon polyp are risk factors for CRC, respectively [18]. However, in two more research conducted in Saudi Arabia, the majority of participants were unaware that family history represented a risk factor [20,23]. Al-Sharif et al. in a similar research indicated that only a small percentage correctly identified the modality for CRC, more than half of the respondents believed that CRC is curable, and less than 20% answered that there is a relationship between IBS and CRC [11]. The majority of participants in a similar study by Ahmed and Alrashidi (83.6%) [20] and Alaqel et al. (72.8%) [16] stated that colonoscopy is the best approach for diagnosing CRC. Furthermore, Almadi et al. [21] in their study recognized that colonoscopy is the most stated tool in more than half (50.5%) of the respondents followed by CT scan (32.7%) and fecal occult blood in nearly one quarter. The colonoscopy was also the most well-known screening method, according to a research carried out in the Tabuk region, Saudi Arabia (31%), and then occult blood test (29%) and CT scan (27%) [24].

More than two-thirds of the respondents revealed that CRC can be cured, but unfortunately, less than one-third indicated that age 50 years and beyond is the ideal time for CRC screening. Similarly, Imran et al. in Jeddah reported that most of the participants (84%) mentioned that CRC is a curable disease [7]. Almadi et al. in Saudi Arabia discovered that the age range of 40-49 years was cited as the best time for screening by 35.1% of respondents [21].

Limitations

Due to the difficulty in getting a well-defined sampling frame, we were unable to draw a random sample from the population, which may have limited the generalizability of the results. The reliance on online platforms as a sampling method may introduce selection bias, and the self-reported data may be subject to recall or social media desirability bias. However, the current research was community-centered, which can be seen as more representative than studies carried out in healthcare settings. The current study's cross-sectional design further limits the use of the causal relationship between the variables.

## Conclusions

Although not perfect, the current analysis demonstrates that there is acceptable public awareness about CRC. We recommend the adoption of education initiatives via mass media and at regular religious events like Jumma in order to increase community awareness and knowledge regarding the risk factors and early screening of CRC for proper management in the northern region of Saudi Arabia.
